# Draft Genome Sequence of *Streptomyces* sp. M1013, a Close Relative of *Streptomyces ambofaciens* and *Streptomyces coelicolor*

**DOI:** 10.1128/genomeA.00643-17

**Published:** 2017-07-20

**Authors:** Drago Haas, Claude Gerbaud, Nevzat Sahin, Jean-Luc Pernodet, Sylvie Lautru

**Affiliations:** a Institute for Integrative Biology of the Cell (I2BC), CEA, CNRS, Université Paris–Sud, Université Paris–Saclay, Gif-sur-Yvette cedex, France; b Department of Biology, Faculty of Art and Science, Ondokyz Mayis University, Kurupelit, Samsun, Turkey

## Abstract

We report the draft genome sequence of *Streptomyces* sp. M1013, a strain isolated from the *Medicago arborea* rhizosphere in Izmir, Turkey. An average nucleotide identity (ANI) analysis reveals that this strain belongs to the same species as *Streptomyces canus* ATCC12647 and is closely related to *Streptomyces ambofaciens* and *Streptomyces coelicolor*.

## GENOME ANNOUNCEMENT

*Streptomyces* bacteria are well known for the wealth of specialized metabolites that they produce (1), and mining sequenced *Streptomyces* genomes for genes typically involved in specialized metabolism usually reveals the presence of 20 to 30 biosynthetic gene clusters (BGCs) (2). When comparing the specialized metabolism of closely related *Streptomyces* species, one can often find a set of BGCs common to all species, and directing the biosynthesis of a “core specialized (secondary) metabolome” (3). In addition to these common BGCs, each species possesses its own array of unique BGCs not shared with the other strains (4), possibly reflecting the differences in their ecological environment.

We are interested in comparing the specialized metabolome of species closely related to *Streptomyces ambofaciens* (5, 6) and *Streptomyces coelicolor* A3(2) (7). We report here the draft genome sequence of *Streptomyces* sp. M1013, a strain that appeared to be closely related to these species, based on its 16S ribosomal DNA sequence. *Streptomyces* sp. M1013 was isolated from a soil sample collected from the *Medicago arborea* rhizosphere in Ege University Botanic Garden in Izmir, Turkey, in July 2005. A paired-end library of the whole genome was constructed and sequenced with GA-IIx (Illumina), generating 52.7 million 38 bp reads that were assembled using Velvet v1.2.10. A total of 43 scaffolds of > 1 kb were obtained. The total size of the assembly was 8,448,284 bp, with a G+C content of 72.0% and a coverage of 224-fold. Coding sequences were automatically predicted using the NCBI Prokaryotic Genome Annotation Pipeline (8). A total of 7,374 protein-coding genes, 65 tRNA, and 18 rRNA genes (6 complete, 12 partial) were found.

The relatedness of *Streptomyces* sp. M1013 with *S. ambofaciens* and *S. coelicolor* was assessed by calculating the average nucleotide identity (ANI) using the JSpeciesWS web server (9). The ANI is 91.58% between *Streptomyces* sp. M1013 and *S. coelicolor* and 87.11% between *Streptomyces* sp. M1013 and *S. ambofaciens* (86.77% between *S. coelicolor* and *S. ambofaciens*), indicating that these strains belong to distinct but closely related species. The ANI is 98.53% between M1013 and *Streptomyces canus* C509 (ATCC12647), indicating that these strains belong to the same species.

Prediction of BGC was carried out using antiSMASH 3.0 (10). A total of 30 BGCs were predicted, including 3 nonribosomal peptide synthetase (NRPS) BGCs and 8 polyketide synthase (PKS) BGCs. BGCs common to the three species include the 2 BGCs directing the biosynthesis of the 2 siderophores coelichelin and desferrioxamine, the 4 BGCs directing the biosynthesis of the terpene derived albaflavenone, geosmin, isorenieratene, and hopene, and the commonly conserved BGCs directing the biosynthesis of melanin, ectoine, and grey spore pigment. In addition, 3 BGCs directing the biosynthesis of putative bacteriocins and lantipeptide, and 2 BGCs containing genes encoding NRPS-independent siderophore (NIS) enzymes are also conserved in the three species.

### Accession number(s).

This whole-genome shotgun project has been deposited in DDBJ/ENA/GenBank under the accession no. MQUH00000000. The version described in this paper is the first version, MQUH00000000.1.
